# Isolation of Novel CreER^T2^-Driver Lines in Zebrafish Using an Unbiased Gene Trap Approach

**DOI:** 10.1371/journal.pone.0129072

**Published:** 2015-06-17

**Authors:** Peggy Jungke, Juliane Hammer, Stefan Hans, Michael Brand

**Affiliations:** Biotechnology Center and Center for Regenerative Therapies Dresden, Dresden University of Technology, Fetscherstrasse 105, 01307 Dresden, Germany; Institute of Molecular and Cell Biology, SINGAPORE

## Abstract

Gene manipulation using the Cre/loxP-recombinase system has been successfully employed in zebrafish to study gene functions and lineage relationships. Recently, gene trapping approaches have been applied to produce large collections of transgenic fish expressing conditional alleles in various tissues. However, the limited number of available cell- and tissue-specific Cre/CreER^T2^-driver lines still constrains widespread application in this model organism. To enlarge the pool of existing CreER^T2^-driver lines, we performed a genome-wide gene trap screen using a *Tol2*-based mCherry-T2a-CreER^T2^ (mCT2aC) gene trap vector. This cassette consists of a splice acceptor and a mCherry-tagged variant of CreER^T2^ which enables simultaneous labeling of the trapping event, as well as CreER^T2^ expression from the endogenous promoter. Using this strategy, we generated 27 novel functional CreER^T2^-driver lines expressing in a cell- and tissue-specific manner during development and adulthood. This study summarizes the analysis of the generated CreER^T2^-driver lines with respect to functionality, expression, integration, as well as associated phenotypes. Our results significantly enlarge the existing pool of CreER^T2^-driver lines in zebrafish and combined with Cre–dependent effector lines, the new CreER^T2^-driver lines will be important tools to manipulate the zebrafish genome.

## Introduction

Zebrafish has become an excellent model system to understand gene function in vertebrate development and disease. Several advantages, such as the optical clarity of its embryos, short generation time and large number of offspring enable for large-scale forward mutagenesis [1–6] screens as well as real-time *in vivo* imaging [7–9]. Furthermore, reverse genetic techniques including morpholino-mediated gene knock-down [10], Targeting Induced Local Lesions IN Genomes (TILLING) [11], and targeted gene modification using engineered endonucleases like TALENs or CRISPR/Cas systems [12–16] allow to interfere with zebrafish gene function. In addition, site-specific recombinases (SSRs), which have been an invaluable tool for altering the mouse and fly genome [17–20], have been successfully applied in zebrafish [21–24]. Cre (Causes recombination of the bacteriophage P1 genome) and other SSRs permit for effective conditional mutagenesis and genetic fate mapping, using a common mechanism of DNA recombination including strand cleavage, exchange and ligation [25–27], which is mediated through defined target sites (loxP sites). To achieve temporal control of the recombination process, ligand-inducible forms have been developed. To this end, ligand-binding domains (LBDs) from homodimeric nuclear receptors, such as the human estrogen receptor (ER), have been used to generate CreER [28] fusions. At the moment, CreER^T2^ shows the best properties in terms of ligand sensitivity and inducible recombination efficiency [29]. Upon administration of Tamoxifen (TAM) or its metabolite 4-hydroxy-Tamoxifen (4-OHT), a conformational change of the LBD mediates translocation of the fusion protein from the cytoplasm into the nucleus and leads to subsequent site-specific recombination. Depending on the nature of the Cre-effector constructs, application of site-specific approaches allows e.g. for cell lineage tracing [22], genetic ablation [30, 31], misexpression studies [32] or conditional gene activity [33–35].

Whereas genome-wide approaches have been conducted to create Cre-effector lines [33–35], the limited number of available cell- and tissue-specific Cre/CreER^T2^-driver lines still restricts its widespread application in zebrafish [36]. Broad expression of Cre/CreER^T2^ can be achieved using the inducible *heat shock cognate 70-kd protein*, *like* (*hsp70l*) [21, 37] or the *ubiquitin b* (*ubb*) promoter [38] and tissue-restricted Cre/CreER^T2^-driver lines have been reported that allow genetic lineage labeling studies or transgene overexpression. However, at present only 59 Cre/CreER^T2^-driver lines have been described in zebrafish (Table 1), whereas more than 2000 driver lines have been created in the mouse model so far [39–41]. Recently, trapping approaches have been applied to produce large collections of transgenic fish expressing conditional alleles in various tissues [34, 35, 42, 43]. Enhancer trapping screens yielded a large library of tissue-specific reporter and driver lines [44]. However, non-specific background expression and also promoter-dependent integration biases reveal limitations of this trapping strategy [45–48]. In addition to enhancer trapping, multiple gene trapping approaches have been applied to dissect the zebrafish genome [5, 33–35, 43]. Gene trapping enables for transgene expression driven by the endogenous promoter and can also be advantageous over transposon-mediated transgenesis using promoter fragments, which often do not faithfully recapitulate the endogenous expression level [22, 49]. In contrast, gene traps fully recapitulate the endogenous gene expression pattern without background expression unless the trapping event interferes with post‐transcriptional regulation of gene expression [49, 50]. Thus in general, gene trapping provides a fast and unbiased method to create tissue-specific driver lines on a large scale basis.

**Table 1:** Summary of currently available Cre/CreER^T2^-driver lines in zebrafish.

Nr.	Cre-driver line	Conditional Cre-driver line	Reference
**1**	***Tg(hsp70l*:*EGFP-cre)ku1***	*** ***	**Thummel R, Burket CT, Brewer JL, Sarras MP, Jr., Li L, et al. (2005) Cre-mediated site-specific recombination in zebrafish embryos. Dev Dyn 233: 1366–1377.**
**2**	***Tg(hsp70l*:*Cre)zdf13***	*** ***	**[Feng H, Langenau DM, Madge JA, Quinkertz A, Gutierrez A, et al. (2007) Heat-shock induction of T-cell lymphoma/leukaemia in conditional Cre/lox-regulated transgenic zebrafish. Br J Haematol 138: 169–175**
**3**	***Tg(zp3*:*cre; krt8*:*rfp)gz14***	*** ***	**[Liu X, Li Z, Emelyanov A, Parinov S, Gong Z (2008) Generation of oocyte-specifically expressed cre transgenic zebrafish for female germline excision of loxP-flanked transgene. Dev Dyn 237: 2955–2962.**
**4**	** **	***Tg(pax2a*:*CreERT2)tud101***	**Hans S, Kaslin J, Freudenreich D, Brand M (2009) Temporally-controlled site-specific recombination in zebrafish. PLoS One 4: e4640**
**5**	** **	***Tg(pax2a*:*CreERT2)tud102***	**Hans S, Kaslin J, Freudenreich D, Brand M (2009) Temporally-controlled site-specific recombination in zebrafish. PLoS One 4: e4640**
**6**	***Tg(-1*.*5ins*:*Cre*,*-*.*58cryaa*:*Venus)s924***	*** ***	**[Hesselson D, Anderson RM, Beinat M, Stainier DY (2009) Distinct populations of quiescent and proliferative pancreatic beta-cells identified by HOTcre mediated labeling. Proc Natl Acad Sci U S A 106: 14896–14901.**
**7**	***Tg(-1*.*5hsp70l*:*Cre)vu297***	*** ***	**[6 Boniface EJ, Lu J, Victoroff T, Zhu M, Chen W (2009) FlEx-based transgenic reporter lines for visualization of Cre and Flp activity in live zebrafish. Genesis 47: 484–491.**
**8**	***Tg(-1*.*8myl7*:*Cre)vu300***	*** ***	**[Boniface EJ, Lu J, Victoroff T, Zhu M, Chen W (2009) FlEx-based transgenic reporter lines for visualization of Cre and Flp activity in live zebrafish. Genesis 47: 484–491.**
**9**	** **	***Tg(-3her4*.*1*:*ERT2-CreERT2)vu298a***	**[Boniface EJ, Lu J, Victoroff T, Zhu M, Chen W (2009) FlEx-based transgenic reporter lines for visualization of Cre and Flp activity in live zebrafish. Genesis 47: 484–491.**
**10**	** **	***Tg(-3her4*.*1*:*ERT2-CreERT2)vu298b***	**[Boniface EJ, Lu J, Victoroff T, Zhu M, Chen W (2009) FlEx-based transgenic reporter lines for visualization of Cre and Flp activity in live zebrafish. Genesis 47: 484–491.**
**11**	** **	***Tg(-3her4*.*1*:*ERT2-CreERT2)vu298c***	**Boniface EJ, Lu J, Victoroff T, Zhu M, Chen W (2009) FlEx-based transgenic reporter lines for visualization of Cre and Flp activity in live zebrafish. Genesis 47: 484–491.**
**12**	** **	***Tg(-3her4*.*1*:*CreERT2)vu299***	**Boniface EJ, Lu J, Victoroff T, Zhu M, Chen W (2009) FlEx-based transgenic reporter lines for visualization of Cre and Flp activity in live zebrafish. Genesis 47: 484–491.**
**13**	*** ***	***Tg(-1myl7*:*ERT2-CreERT2-IRES-mCherry)be1***	**Jopling C, Sleep E, Raya M, Marti M, Raya A, et al. (2010) Zebrafish heart regeneration occurs by cardiomyocyte dedifferentiation and proliferation. Nature 464: 606–609.**
**14**	** **	***Tg(Cau*.*Tuba1a*:*CreERT2*,*Cau*.*Tuba1a*:*CFP)mi19/+***	**Ramachandran R, Reifler A, Parent JM, Goldman D (2010) Conditional gene expression and lineage tracing of tuba1a expressing cells during zebrafish development and retina regeneration. J Comp Neurol 518: 4196–4212**
**15**	***Tg(kdrl*:*Cre)s898***	*** ***	**Bertrand JY, Chi NC, Santoso B, Teng S, Stainier DY, et al. (2010) Haematopoietic stem cells derive directly from aortic endothelium during development. Nature 464: 108–111.**
**16**	** **	***Tg(cryaa*:*DsRed*,*-5*.*1myl7*:*CreERT2)pd12***	**Liu J, Bressan M, Hassel D, Huisken J, Staudt D, et al. (2010) A dual role for ErbB2 signaling in cardiac trabeculation. Development 137: 3867–3875.**
**17**	** **	***Tg(-14*.*8gata4*:*ERT2-CreERT2)pd39***	**Kikuchi K, Holdway JE, Werdich AA, Anderson RM, Fang Y, et al. (2010) Primary contribution to zebrafish heart regeneration by gata4(+) cardiomyocytes. Nature 464: 601–605.**
**18**	***Tg(actb2*:*Cerulean-Cre)ct5000***	*** ***	**Trinh le A, Hochgreb T, Graham M, Wu D, Ruf-Zamojski F, et al. (2011) A versatile gene trap to visualize and interrogate the function of the vertebrate proteome. Genes Dev 25: 2306–2320.**
**19**	***Tg(lmo2*:*Cre)rj5***	*** ***	**Zhou T, Wang L, Zhu KY, Dong M, Xu PF, et al. (2011) Dominant-negative C/ebpalpha and polycomb group protein Bmi1 extend short-lived hematopoietic stem/progenitor cell life span and induce lethal dyserythropoiesis. Blood 118: 3842–3852.**
**20**	***TgBAC(-25ltbp3*:*TagRFP-Cre)fb1***	*** ***	**Zhou T, Wang L, Zhu KY, Dong M, Xu PF, et al. (2011) Dominant-negative C/ebpalpha and polycomb group protein Bmi1 extend short-lived hematopoietic stem/progenitor cell life span and induce lethal dyserythropoiesis. Blood 118: 3842–3852.**
**21**	***Tg(ela3l*:*Cre*,*cryaa*:*Venus)s932***	*** ***	**Hesselson D, Anderson RM, Stainier DY (2011) Suppression of Ptf1a activity induces acinar-to-endocrine conversion. Curr Biol 21: 712–717.**
**22**	*** ***	***Tg(-3*.*5ubb*:*CreERT2*, *myl7*:*EGFP)cz1702***	**Mosimann C, Kaufman CK, Li P, Pugach EK, Tamplin OJ, et al. (2011) Ubiquitous transgene expression and Cre-based recombination driven by the ubiquitin promoter in zebrafish. Development 138: 169–177.**
**23**	** **	***Tg(EPV*.*Tp1-Ocu*.*Hbb2*:*CreERT2)jh12***	**Wang Y, Rovira M, Yusuff S, Parsons MJ (2011) Genetic inducible fate mapping in larval zebrafish reveals origins of adult insulin-producing beta-cells. Development 138: 609–617.**
**24**	** **	***Tg(actb2*:*GFP-CreERT2)jh29***	**Wang Y, Rovira M, Yusuff S, Parsons MJ (2011) Genetic inducible fate mapping in larval zebrafish reveals origins of adult insulin-producing beta-cells. Development 138: 609–617.**
**25**	** **	***TgBAC(cryaa*:*EGFP*,*tcf21*:*CreERT2)pd42***	**Kikuchi K, Gupta V, Wang J, Holdway JE, Wills AA, et al. (2011) tcf21+ epicardial cells adopt non-myocardial fates during zebrafish heart development and regeneration. Development 138: 2895–2902.]**
**26**	** **	***Tg(cryaa*:*DsRed*,*-5*.*1myl7*:*CreERT2)pd10***	**Kikuchi K, Gupta V, Wang J, Holdway JE, Wills AA, et al. (2011) tcf21+ epicardial cells adopt non-myocardial fates during zebrafish heart development and regeneration. Development 138: 2895–2902.**
**27**	** **	***Tg(Ola*.*Sp7*:*CreERT2-2A-mCherry)tud8***	**Knopf F, Hammond C, Chekuru A, Kurth T, Hans S, et al. (2011) Bone regenerates via dedifferentiation of osteoblasts in the zebrafish fin. Dev Cell 20: 713–724.**
**28**	** **	***Tg(hsp70l*:*mCherry*,*CreERT2)tud104***	**Hans S, Freudenreich D, Geffarth M, Kaslin J, Machate A, et al. (2011) Generation of a non-leaky heat shock-inducible Cre line for conditional Cre/lox strategies in zebrafish. Dev Dyn 240: 108–115.**
**29**	** **	***Tg(hsp70l*:*mCherry*,*CreERT2)tud105***	**Hans S, Freudenreich D, Geffarth M, Kaslin J, Machate A, et al. (2011) Generation of a non-leaky heat shock-inducible Cre line for conditional Cre/lox strategies in zebrafish. Dev Dyn 240: 108–115.**
**30**	** **	***Tg(her4*.*1*:*mCherry*,*CreERT2)tud106***	**Kroehne V, Freudenreich D, Hans S, Kaslin J, Brand M (2011) Regeneration of the adult zebrafish brain from neurogenic radial glia-type progenitors. Development 138: 4831–4841.**
**31**	***TgBAC(dbx1b*:*Cre-mCherry)nns13a***	*** ***	**[Satou C, Kimura Y, Higashijima S (2012) Generation of multiple classes of V0 neurons in zebrafish spinal cord: progenitor heterogeneity and temporal control of neuronal diversity. J Neurosci 32: 1771–1783.**
**32**	***Tg(-2*.*8fabp10a*:*Cre*,*cryaa*:*Venus)s955***	*** ***	**Ni TT, Lu J, Zhu M, Maddison LA, Boyd KL, et al. (2012) Conditional control of gene function by an invertible gene trap in zebrafish. Proc Natl Acad Sci U S A.**
**33**	***Tg(Mmu*.*Sox10-Mmu*.*Fos*:*Cre)zf384***	*** ***	**[Kague E, Gallagher M, Burke S, Parsons M, Franz-Odendaal T, et al. (2012) Skeletogenic fate of zebrafish cranial and trunk neural crest. PLoS One 7: e47394.**
**34**	***Tg(-4*.*7sox10*:*Cre)ba73***	*** ***	**Rodrigues FS, Doughton G, Yang B, Kelsh RN (2012) A novel transgenic line using the Cre-lox system to allow permanent lineage-labeling of the zebrafish neural crest. Genesis 50: 750–757.**
**35**	***Tg(-4*.*7sox10*:*Cre)ba74***	*** ***	**Hammond CL, Moro E (2012) Using transgenic reporters to visualize bone and cartilage signaling during development in vivo. Front Endocrinol (Lausanne) 3: 91.**
**36**	***Tg(-4*.*7sox10*:*Cre)ba101***	*** ***	**Rodrigues FS, Doughton G, Yang B, Kelsh RN (2012) A novel transgenic line using the Cre-lox system to allow permanent lineage-labeling of the zebrafish neural crest. Genesis 50: 750–757.**
**37**	** **	***Tg(hsp70l*.*1*:*mcherry*,*CreERT2)jk67***	**Yoshinari N, Ando K, Kudo A, Kinoshita M, Kawakami A (2012) Colored medaka and zebrafish: transgenics with ubiquitous and strong transgene expression driven by the medaka beta-actin promoter. Dev Growth Differ 54: 818–828.**
**38**	** **	***Tg(Ola*.*Sp7*:*TagBFP*,*CreERT2)pd45/+***	**Singh SP, Holdway JE, Poss KD (2012) Regeneration of amputated zebrafish fin rays from de novo osteoblasts. Dev Cell 22: 879–886.**
**39**	** **	***Tg(dusp6*:*CreERT2*,*myl7*:*ECFP)b1230***	**Stewart S, Stankunas K (2012) Limited dedifferentiation provides replacement tissue during zebrafish fin regeneration. Dev Biol 365: 339–349.**
**40**	***TgBAC(-25ltbp3*:*TagRFP-Cre)fb1***	*** ***	**Guner-Ataman B, Paffett-Lugassy N, Adams MS, Nevis KR, Jahangiri L, et al. (2013) Zebrafish second heart field development relies on progenitor specification in anterior lateral plate mesoderm and nkx2.5 function. Development 140: 1353–1363.**
**41**	***Tg(kop*:*Cre-UTRnanos3*,*CMV*:*EGFP)ihb7***	*** ***	**Xiong F, Wei ZQ, Zhu ZY, Sun YH (2013) Targeted expression in zebrafish primordial germ cells by Cre/loxP and Gal4/UAS systems. Mar Biotechnol (NY) 15: 526–539.**
**42**	** **	***TgBAC(nkx2*.*5*:*ERT2-CreERT2)fb8***	**Guner-Ataman B, Paffett-Lugassy N, Adams MS, Nevis KR, Jahangiri L, et al. (2013) Zebrafish second heart field development relies on progenitor specification in anterior lateral plate mesoderm and nkx2.5 function. Development 140: 1353–1363.**
**43**	***Tg(sox10*:*Cre*,*myl7*:*EGFP)sq5***	*** ***	**Ho Lee RT, Thiery JP, Carney TJ (2013) Dermal fin rays and scales derive from mesoderm, not neural crest. Curr Biol 23: R336-337.**
**44**	***Tg(tbx6l*:*Cre*,*myl7*:*EGFP)sq6***	*** ***	**Ho Lee RT, Thiery JP, Carney TJ (2013) Dermal fin rays and scales derive from mesoderm, not neural crest. Curr Biol 23: R336-337.**
**45**	** **	***Tg(tbx6l*:*CreERT2*,*myl7*:*EGFP)sq7***	**Ho Lee RT, Thiery JP, Carney TJ (2013) Dermal fin rays and scales derive from mesoderm, not neural crest. Curr Biol 23: R336-337.**
**46**	*** ***	***Tg(sox10*:*CreERT2*,*myl7*:*GFP)t007***	**Mongera A, Singh AP, Levesque MP, Chen YY, Konstantinidis P, et al. (2013) Genetic lineage labeling in zebrafish uncovers novel neural crest contributions to the head, including gill pillar cells. Development 140: 916–925.**
**47**	***Tg2(hsp70l*:*Cre)a134***	*** ***	**Pan YA, Freundlich T, Weissman TA, Schoppik D, Wang XC, et al. (2013) Zebrabow: multispectral cell labeling for cell tracing and lineage analysis in zebrafish. Development 140: 2835–2846.**
**48**	** **	***Tg(amhc*:*CreERT2)sd20***	**Zhang R, Han P, Yang H, Ouyang K, Lee D, et al. (2013) In vivo cardiac reprogramming contributes to zebrafish heart regeneration. Nature 498: 497–501.**
**49**	** **	***Tg(pax2a*:*CreERT2)tud110***	**Hans S, Irmscher A, Brand M (2013) Zebrafish Foxi1 provides a neuronal ground state during inner ear induction preceding the Dlx3b/4b-regulated sensory lineage. Development 140: 1936–1945.**
**50**	***Tg(LOXP-CMV*:*Cre*,*CMV*:*EGFP)***	*** ***	**Lin HJ, Lee SH, Wu JL, Duann YF, Chen JY (2013) Development of Cre-loxP technology in zebrafish to study the regulation of fish reproduction. Fish Physiol Biochem 39: 1525–1539.**
**51**	***TgBAC(gsx1*:*Cre)***	*** ***	**Satou C, Kimura Y, Hirata H, Suster ML, Kawakami K, et al. (2013) Transgenic tools to characterize neuronal properties of discrete populations of zebrafish neurons. Development 140: 3927–3931.**
**52**	***Tg(myl7*:*YFP-Cre)***	*** ***	**Ding Y, Liu W, Deng Y, Jomok B, Yang J, et al. (2013) Trapping cardiac recessive mutants via expression-based insertional mutagenesis screening. Circ Res 112: 606–617.**
**53**	*** ***	***Tg(fabp10a*:*CreERT2)***	**Choi TY, Ninov N, Stainier DY, Shin D (2013) Extensive conversion of hepatic biliary epithelial cells to hepatocytes after near total loss of hepatocytes in zebrafish. Gastroenterology 146: 776–788.**
**54**	*** ***	***Tg(TP1*:*CreRT2)***	**Ninov N, Hesselson D, Gut P, Zhou A, Fidelin K, et al. (2013) Metabolic regulation of cellular plasticity in the pancreas. Curr Biol 23: 1242–1250.**
**55**	*** ***	***Tg(krt4*:*CreERT2*,*myl7*:*EGFP)***	**Lee RT, Asharani PV, Carney TJ (2014) Basal keratinocytes contribute to all strata of the adult zebrafish epidermis. PLoS One 9: e84858.**
**56**	*** ***	***Tg(krtt1c19e*:*CreERT2*,*myl7*:*EGFP)***	**Lee RT, Asharani PV, Carney TJ (2014) Basal keratinocytes contribute to all strata of the adult zebrafish epidermis. PLoS One 9: e84858.**
**57**	*** ***	***Tg(krtt1c19e*:*CreERT2)***	**Fischer B, Metzger M, Richardson R, Knyphausen P, Ramezani T, et al. (2014) p53 and TAp63 promote keratinocyte proliferation and differentiation in breeding tubercles of the zebrafish. PLoS Genet 10: e1004048.**
**58**	*** ***	***Tg(krt4*:*CreERT2)***	**Fischer B, Metzger M, Richardson R, Knyphausen P, Ramezani T, et al. (2014) p53 and TAp63 promote keratinocyte proliferation and differentiation in breeding tubercles of the zebrafish. PLoS Genet 10: e1004048.**
**59**	** **	***Tg(kdrl*:*Cre-ERT2)***	**Zhao L, Borikova AL, Ben-Yair R, Guner-Ataman B, MacRae CA, et al. (2014) Notch signaling regulates cardiomyocyte proliferation during zebrafish heart regeneration. Proc Natl Acad Sci U S A 111: 1403–1408.**

Cre- and CreER^T2^-driver lines available in zebrafish are listed in chronological order including citation.

**Fig 1:**
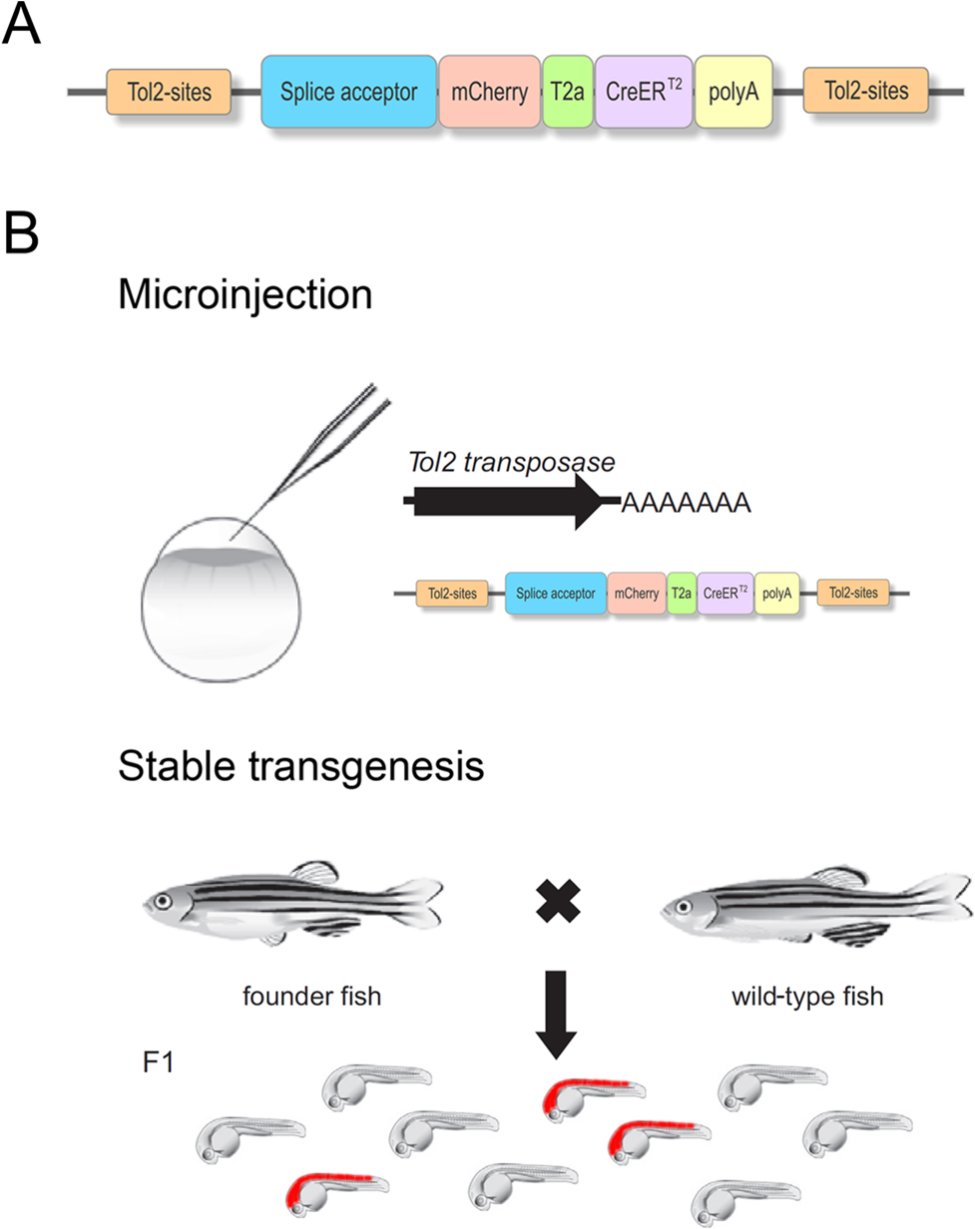
Generation of CreER^T2^-driver lines via gene trapping. (A) *Tol2*-based mCT2aC gene trap vector comprising a splice acceptor, the mCherry sequence separated from CreER^T2^ via T2a. (B) Work schedule for the generation of CreER^T2^-driver lines using a gene trap approach.

To expand the existing pool of CreER^T2^-driver lines in zebrafish, we performed a genome-wide gene trap screen, using a vector that reports gene expression via the fluorescence protein mCherry and simultaneously drives CreER^T2^ under the endogenous promoter. To this end, we applied the mCherry-T2a-CreER^T2^ (mCT2aC) gene trap construct consisting of a splice acceptor (SA) and a single open reading frame coding for mCherry and CreER^T2^, separated by the viral T2a peptide sequence (Fig 1A) [21, 24, 49]. In total, we used three different mCT2aC gene trap vectors, yielding 27 novel CreER^T2^-driver lines expressing in various tissues in the developing and adult zebrafish. All lines were analyzed with respect to integration, expression, functionality and potential phenotype caused by the insertion.

## Material and Methods

### Cloning of the *pTol-SA*_*x*_ *-mCT2aC* gene trap cassettes

To generate the rabbit *β-globin* SA containing plasmid *pTol-SA1-mCT2aC*, the mCherry-T2a-CreER^T2^ cassette (mCT2aC) was cloned into the *Tol2* transposon-based gene trap vector *pT2KSAG* plasmid [5]. To create the *pTol-SA2-mCT2aC* plasmid the zebrafish *bcl2* SA was amplified from the *T2BGS* plasmid [51] using the following primers flanked by the indicated restriction sites: Bcl2-for (Apa1) atatGGGCCCtagcagtttcatgcaccatagaccgc; Egfp r4-rev (Fse1) atatGGCCGGCCgatgggcaccaccccggtga that allowed substitution of the SA1 of the *pTol-SA1-mCT2aC* plasmid. Similarly, to generate the *pTol-SA3-mCT2aC* plasmid the zebrafish *gata6* SA was amplified from 24 hpf wild-type AB cDNA using the following primers flanked by the indicated restriction sites: GATA6-for (Apa1) atatGGGCCCtataagtagactgttaggttggggttaggat; GATA6-5’-rev (Fse1) atatGGCCGGCCcctggatcagagcagagaatgtccgtg that allowed substitution of the SA1 of the *pTol-SA1-mCT2aC* plasmid.

### Zebrafish husbandry, germ line transformation and screening of F1 progeny

Zebrafish embryos were obtained by natural spawnings of adult wild-type AB fish maintained at 28.5°C on a 14-hr light, 10-hr dark cycle and staged as described [52, 53]. For germ line transformation, 30 pg plasmid DNA and 30 pg transposase mRNA were injected into fertilized eggs (F0), raised to adulthood and crossed to wild-type AB fish as previously described [5]. To identify transgenic carriers, F1 embryos were screened for mCherry under a fluorescent microscope (Olympus MVX10) at various developmental stages (1–5 dpf). mCherry positive embryos were raised and re-identified in the F2 generation.

### Insertion mapping using 5’RACE and inverse PCR (iPCR)

Mapping of insertions was done by 5’RACE on the cDNA level. RNA was isolated from 24 to 48 hpf mCherry positive 10–15 embryos using Trizol (Ambion, Life Technologies) according to the manufacturer’s protocol. 5’RACE was performed according to the manufacturer’s protocol of the SMARTer RACE cDNA Amplification Kit (Clontech) with the following primers: (mcherry rev 5’- AGTTCATCACGCGCTCCCACTTGAAGCC and mcherry rev 2 5’- CGTAGGCCTTGGAGCCGTAC (as nested primer)). Mapping of gene trap insertions on DNA level was done by inverse PCR as previously published [54] with modification of primers (1st PCR: Tol for1 3‘ TTTACTCAAGTAAGATTCTAG; Tol rev1 3‘ CTCCATTAAAATTGTACTTG; Tol for1 5‘ CTTGAGTACAATTAAAAATCAATAC; Tol rev1 5‘ GTAAAAATCCCCAAAAATAATAC; 2^nd^ PCR: Tol for2 3‘ ACTTGTACTTTCACTTGAGTA; Tol rev2 3‘ GCAAGAAAGAAAACTAGAGA; Tol for2 5‘ CTCCTTACAATTTTATTTACAGTC; Tol rev2 5‘ GTAAAATTACTCAAGTACTTTACACC (communication with J.Bessa).

### Expression analysis of transgenic lines

Expression patterns of respective CreER^T2^-driver lines were analyzed using native mCherry fluorescence as well as *in situ* hybridization (ISH) analysis for CreER^T2^. Probe synthesis and ISH was performed essentially as previously described [55, 56] using the vector pCs2+-CreER^T2^ [22]. Native mCherry fluorescence and stainings were analyzed using a Zeiss Axiophot 2 or an Olympus MVX10 microscope.

### Pharmacological treatments and functionality assay

For Tamoxifen (TAM) and 4-hydroxy-Tamoxifen (4-OHT) (Sigma, St. Louis, MO;T5648 and H7904) treatments, a 50 mM and 25 mM stock solution was made in DMSO and ethanol and stored at -20°C. To test the functionality of the respective CreER^T2^-driver lines, the individual CreER^T2^-driver line was crossed with the Cre-dependent reporter line *Tg(hsp70l*:*loxP-DsRed-loxP-EGFP)* which expresses DsRed2 under the control of the ubiquitous, temperature inducible *hsp70l* promoter, but switches permanently to EGFP after a successful recombination event [24]. For embryonic treatment, progeny of this cross were exposed to 5 μM TAM from 6 hpf to 24 hpf to elicit recombination, heat shocked at 24 hpf for 1 hour to activate reporter expression and analyzed at 28 hpf. CreER^T2^-driver lines with an onset of CreER^T2^ beyond 24 hpf were exposed to 5 μM TAM from 36 hpf to 48 hpf, heat shocked at 48 hpf and analyzed at 52 hpf. For larval treatment, progeny of this cross were exposed to 1 μM 4-OHT from 80 hpf to 96 hpf to elicit recombination, heat shocked at 96 hpf for 1 hour to activate reporter expression and analyzed at 100 hpf.

### Nomenclature guidelines

CreER^T2^-driver lines are designated based on ZFIN nomenclature. Full names are presented as e.g. Gt(SA3-mCT2aC)tud37, which reflects the utilized splice acceptor (SA3), the mCherry-T2a-CreER^T2^-cassette (mCT2aC) and the individual line designation number (e.g. 37) with respect to our institute (tud). To increase readability CreER^T2^-driver lines are abbreviated throughout the manuscript (e.g. CreER^T2^-driver line Gt(SA3-mCT2aC)tud37: tud37Gt).

### Ethics statement

All experiments were conducted in accordance with the Animal Welfare Act and with permission of the federal authorities (Landesdirektion Sachsen AZ 24–9168.11-1/2013-14, Germany). Moreover, according to the EU Directive 2010/63/EU on the protection of animals used for scientific purposes, early life-stages of zebrafish are not protected as animals until the stage of being capable of independent feeding (5 days post fertilization). In this study the experiments did not exceed an exposure time of 4 days post fertilization, thus, the zebrafish utilized were not capable of independent feeding and not protected as animals according to the EU Directive mentioned above.

## Results

### The mCT2aC gene trap screen

**Fig 2:**
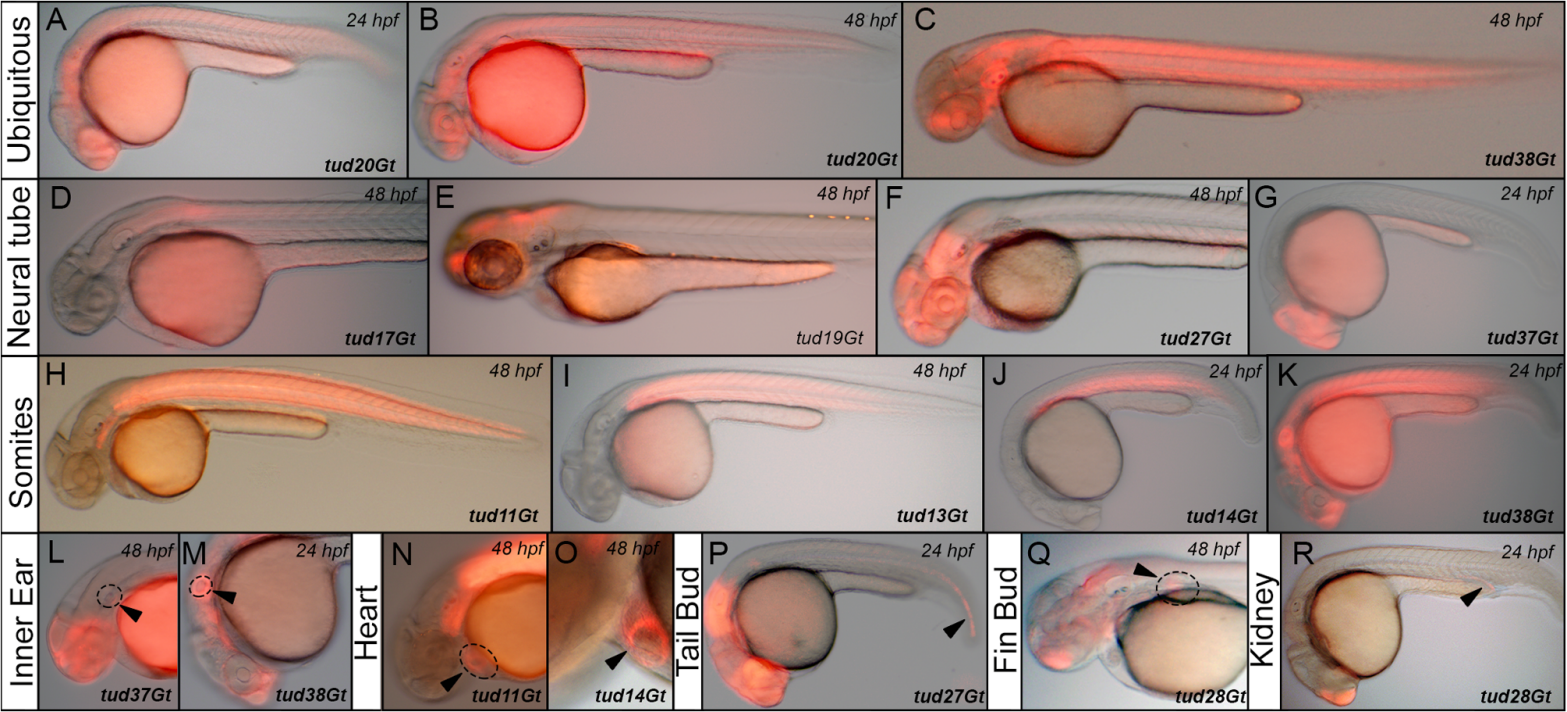
Native mCherry expression of CreER^T2^-driver lines in a variety of embryonic tissues. (A-C) Ubiquitous mCherry expression in tud20Gt at 24 and 48 hpf, and tud38Gt at 48 hpf. (D-G) CreER^T2^-driver lines expressing mCherry in restricted patterns of the neural tube: (D) hindbrain and spinal cord expression in tud17Gt at 48 hpf, (E-G) fore-, mid- and hindbrain expression in tud19Gt and tud27Gt at 48 hpf and fore- and midbrain expression in tud37Gt at 24 hpf. (H-K) Somitic mCherry expression in tud11Gt and tud13Gt at 48 hpf as well as in tud14Gt and tud38Gt at 24 hpf. (L, M) mCherry expression in the developing inner ear in tud37Gt at 48 hpf and tud38Gt at 24 hpf. (N, O) CreER^T2^-driver lines expressing mCherry in the embryonic heart in tud11Gt and tud14Gt at 48 hpf. (P) mCherry expression in the tail bud in tud27Gt at 24 hpf. (Q) Fin bud expression in tud28Gt at 48 hpf. (R) CreER^T2^-driver line tud28Gt shows mCherry expression in the kidney anlagen at 24 hpf; Bold letters indicate CreER^T2^-driver lines with known gene trap integrations.

In order to obtain a wide variety of CreER^T2^-driver lines a gene trap approach was chosen using a vector containing a splice acceptor (SA) and a mCherry-tagged variant of CreER^T2^ (consisting of a single open reading frame coding for mCherry and CreER^T2^ separated by the viral T2A peptide sequence) followed by a polyadenylation (p(A)) signal (Fig 1A). To avoid any SA site-specific integration bias three different trapping vectors were generated containing different SA sites [22] [49] (*pTol-SA1-mCT2aC*: rabbit *β-globin* SA; *pTol-SA2-mCT2aC*: zebrafish *bcl2* SA; *pTol-SA3-mCT2aC*: zebrafish *gata6* SA). Upon random integration of the gene trap vector into an endogenous locus, a fusion between the 5’-located exons and the gene trap cassette is generated. The p(A) signal within the gene trap cassette mediates transcriptional termination, resulting in a truncation of the endogenous gene sequence. The separation of the bicistronic message using T2a allows equimolar production of both mCherry and CreER^T2^. Depending on the insertion site, information on the subcellular localization of the trapped protein is provided by the mCherry tag. To generate mCherry-tagged CreER^T2^-driver lines, we injected one-cell-stage wild-type embryos with *Tol2* transposase mRNA together with the respective gene trap construct. Injected fish were raised and outcrossed to wild-type fish as previously described [5]. The resulting F1 embryos were examined under a fluorescent microscope at various developmental stages (1–5 dpf) and mCherry positive embryos were selected and raised (Fig 1B). Co-transmission of gene trap integrations was resolved in subsequent generations and only carriers of single insertion events were raised further. In total, 1479 fish were screened for the different gene trap constructs (*pTol-SA1-mCT2aC*: 1034 fish; *pTol-SA2-mCT2aC*: 177 fish; *pTol-SA3-mCT2aC*: 268 fish) yielding trapping rates of 8,7%, 14,1% and 11,6% respectively, which is consistent with previous data [5, 35, 57]. Out of 148 insertions, 42 were selected and established as stable transgenic lines and further analyzed including a recombination functionality assay, embryonic expression profiling using CreER^T2^ ISH and native mCherry fluorescence, transgene mapping and investigation of integration-related phenotypes. In all 42 transgenic lines, inheritance rates of approximately 50% were consistent with the ratio of Mendelian segregation and observed through additional generations indicating integration into only one active locus per line. Southern blot analysis to determine multi-copy integration at the active locus or the overall copy number integrated in the genomes was not performed. A variety of mCherry expression patterns were observed in transgenic F1 embryos, indicating that the mCT2aC cassette was inserted into various loci in the genome and is expressed under the control of various endogenous promoters. In addition to ubiquitous expression (Fig 2A–2C) native mCherry expression could be detected e.g. in the anlagen of the neural tube (Fig 2D–2G), somites (Fig 2H–2K), inner ear (Fig 2L and 2M), heart (Fig 2N and 2O), tail bud (Fig 2P), fin bud (Fig 2Q) or kidney (Fig 2R).

Taken together, we find that gene trapping provides a rapid and unbiased method to create tissue-specific CreER^T2^-driver lines expressing in multiple tissues of the developing zebrafish.

### Functional analysis of mCT2aC gene trap lines

**Fig 3:**
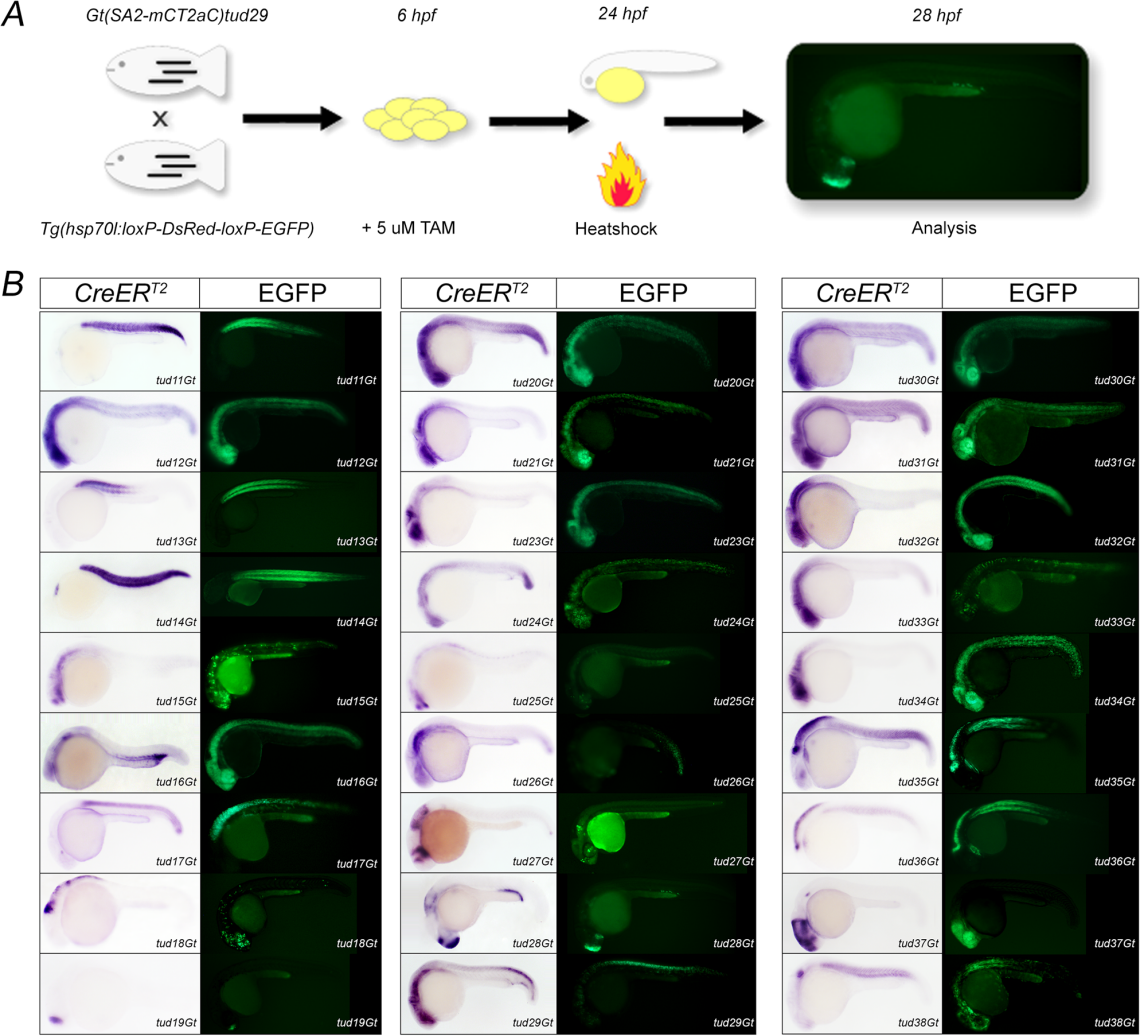
Identification of functional CreER^T2^-driver lines. (A) Scheme of the embryonic functionality assay. (B) 27 CreER^T2^-driver lines are shown with respect to CreER^T2^ expression at 24 hpf (CreER^T2^) and the respective embryonic functionality assay indicated by native EGFP fluorescence.

To demonstrate the functionality of each CreER^T2^-driver line, we applied a standardized functionality assay [49]. In this assay the respective CreER^T2^-driver line (e.g. tud28Gt) was crossed to the Cre-dependent reporter line *Tg(hsp70l*:*loxP-DsRed-loxP-EGFP)* which expresses DsRed2 under the control of the ubiquitous, temperature inducible *hsp70l* promoter, but switches permanently to EGFP after a successful recombination event [24]. For embryonic treatment, progeny of this cross were exposed to 5 μM TAM from 6 hpf to 24 hpf to elicit recombination, heat shocked at 24 hpf for 1 hour to activate reporter expression and analyzed at 28 hpf (Fig 3A). Analysis of the selected 42 CreER^T2^-driver lines using this functionality assay showed successful recombination in 27 CreER^T2^-driver lines, or 64% of the gene trap lines (Fig 3B). In contrast, 15 CreER^T2^-driver lines, or 36% of the tested lines, did not show any successful recombination indicated by lack of green fluorescence (data not shown). In most cases, positive recombination (shown as native EGFP fluorescence) recapitulated the endogenous expression pattern detected by the CreER^T2^ ISH signal. For example, tud11Gt shows somitic CreER^T2^ expression at 24 hpf and native EGFP fluorescence is present in the same tissue at 28 hpf. Other examples are represented by CreER^T2^-driver lines tud17Gt or tud19Gt, where both endogenous CreER^T2^ expression and native EGFP fluorescence was observed in the spinal cord or telencephalon, respectively. However, the functionality assay also revealed positive recombination events in tissues devoid of CreER^T2^-expression at the selected time point, which most likely represent lineage tracings of cells expressing CreER^T2^ at earlier stages of development. For example, tud21Gt and tud23Gt show CreER^T2^ expression at 24 hpf only in the brain, whereas native EGFP fluorescence can be observed in the entire neural tube at 28 hpf. Similarly, CreER^T2^ expression in tud32Gt can only be detected in the developing brain at 24 hpf but native EGFP fluorescence is present in the entire neural tube as well as the somites.

**Fig 4:**
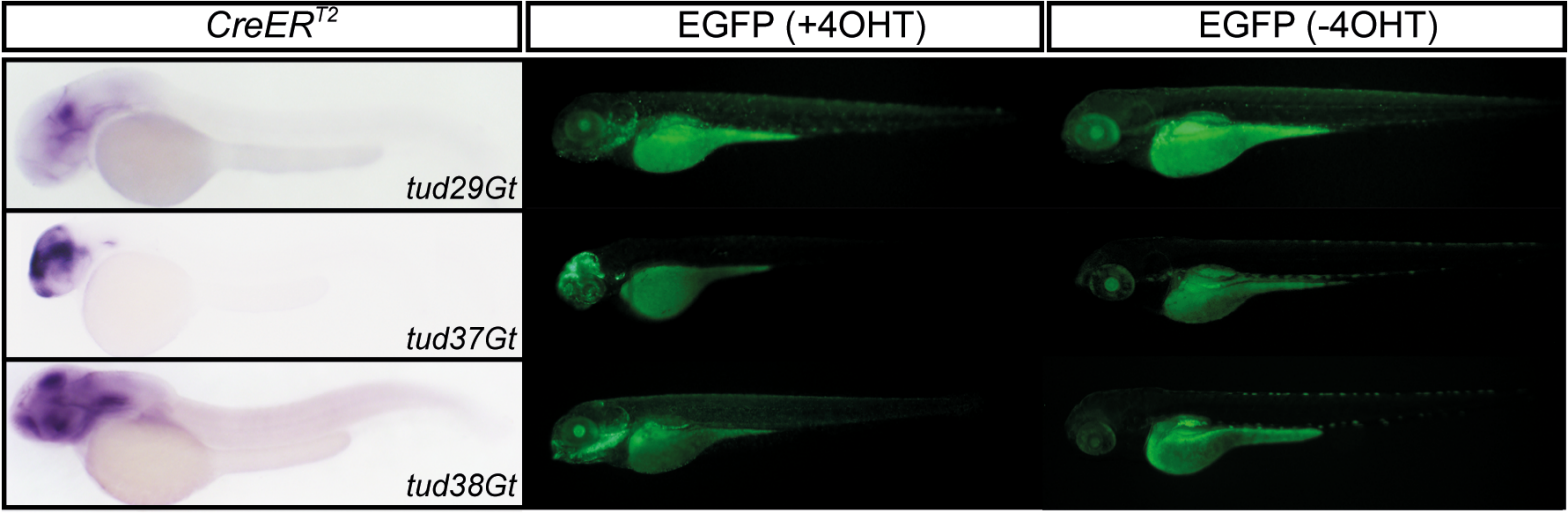
Functionality of CreER^T2^-driver lines at larval stages. tud29Gt, tud37Gt and tud38Gt are shown with respect to CreER^T2^ expression at 48 hpf (CreER^T2^) and EGFP fluorescence in the presence and absence of 4-hydroxy-Tamoxifen (4-OHT) at 100 hpf.

In order to test recombination at larval stages we randomly selected 5 CreER^T2^-driver lines (tud12Gt, tud29Gt, tud31Gt, tud37Gt, tud38Gt) and repeated the cross to the before mentioned Cre-dependent reporter line. Because treatment with 5 μM TAM from 80 hpf to 96 hpf followed by heat shock resulted in severe developmental abnormalities, we applied 1 μM 4-hydroxy-Tamoxifen (4-OHT) prior to heat treatment which did not cause any change in proper development. Analysis of tud12Gt and tud31Gt did not result in any successful recombination under these conditions (data not shown). In contrast, successful recombination reported by robust and strong EGFP fluorescence was observed in tud29Gt, tud37Gt and tud38Gt recapitulating the endogenous expression pattern detected by CreER^T2^ ISH (Fig 4). Furthermore, no EGFP expression could be detected in the absence of 4-OHT indicating tight regulation of CreER^T2^ in the respective CreER^T2^-driver lines and absence of any non-conditional recombination (leakiness).Taken together, our analysis revealed that the new gene trap lines are functional CreER^T2^-driver lines allowing for CreER^T2^-mediated transactivation in various tissues at various developmental stages. However, as it has been previously shown that recombination depends on the expression strength of CreER^T2^ [22] TAM or 4-OHT conditions need to be tested and optimized for each CreER^T2^ driver line.

### Molecular characterization of CreER^T2^-driver lines

In order to map the gene trapping events, 5’RACE of mCherry positive embryos was performed. If insertions could not be identified with 5’RACE, inverse PCR was carried out, which has been successfully applied previously [33]. In total 17 (63%) out of 27 functional CreER^T2^-driver lines could be mapped. 11 traps (65%) were identified using 5’RACE and 6 traps (35%) by inverse PCR. The remaining 10 integrations (27%) were inconclusive, consistent with observations by Kawakami and colleagues, who reported that only 50–70% of *Tol2*-integrations can be mapped using 5’RACE [5]. Molecularly identified CreER^T2^-driver lines are summarized in Table 2, including gene trap integration, NCBI gene ID, linkage group as well as insertion site. Transgene mapping revealed vector integration into genes involved in a variety of biological processes and protein classes, such as cytoskeletal proteins (1 gene), extracellular matrix proteins (1 gene), signaling receptors (1 gene), transcription factors (5 genes) or nucleic acid binding proteins (6 genes) (source PANTHER database http://www.pantherdb.org [58]). Consistent with previous reports, no integration bias into any chromosomal location could be detected for the *Tol2* transposable system [59, 60]. However, most of the gene trap integrations are biased towards the 5’ end of genes. 12 of the mapped insertions integrated into the 5’UTR or exon/intron 1 of the trapped gene. In tud25Gt, tud27Gt and tud38Gt the gene trap cassette was inserted into intron 2 or intron 4 of genes with 19 exons, respectively. Only in tud34Gt and tud37Gt vector integration was observed into the 3’ end of the genes.

**Table 2:** Molecular identification of functional CreER^T2^-driver lines.

CreER^T2^-driver line	Gene trap integration	NCBI Gene ID	Linkage group	Insertion site
***Gt(SA1-mCherry-T2A-CreERT2)tud11Gt***	***ptk2*.*2***	**386705**	**19**	**5'UTR**
***Gt(SA1-mCherry-T2A-CreERT2)tud12Gt***	***mapre1a***	**334135**	**8**	**5'UTR**
***Gt(SA1-mCherry-T2A-CreERT2)tud13Gt***	***pvalb1***	**402805**	**3**	**5'UTR**
***Gt(SA1-mCherry-T2A-CreERT2)tud15Gt***	***klhl17***	**336187**	**23**	**Intron 1**
***Gt(SA1-mCherry-T2A-CreERT2)tud16Gt***	***LOC100002938***	**100002938**	**12**	**Intron 1**
***Gt(SA1-mCherry-T2A-CreERT2)tud17Gt***	***hoxb1b***	**30374**	**2**	**5'UTR**
***Gt(SA1-mCherry-T2A-CreERT2)tud18Gt***	***msxc***	**30526**	**13**	**Exon 1**
***Gt(SA1-mCherry-T2A-CreERT2)tud20Gt***	***si*:*ch73-248e17*.*1***	**Not identified**	**22**	**5'UTR**
***Gt(SA1-mCherry-T2A-CreERT2)tud21Gt***	***baz2ba***	**561095**	**6**	**Intron 1**
***Gt(SA1-mCherry-T2A-CreERT2)tud25Gt***	***vldlr***	**393897**	**10**	**Intron 2**
***Gt(SA2-mCherry-T2A-CreERT2)tud27Gt***	***epha7***	**562195**	**20**	**Intron 4**
***Gt(SA2-mCherry-T2A-CreERT2)tud28Gt***	***si*:*ch211-263k4*.*2***	**796720**	**8**	**Intron 1**
***Gt(SA2-mCherry-T2A-CreERT2)tud29Gt***	***ccdc102a***	**325319**	**7**	**Intron 1**
***Gt(SA3-mCherry-T2A-CreERT2)tud30Gt***	***srsf1b***	**393565**	**21**	**Intron 1**
***Gt(SA3-mCherry-T2A-CreERT2)tud34Gt***	***ywhaba***	**323055**	**6**	**3'UTR**
***Gt(SA3-mCherry-T2A-CreERT2)tud37Gt***	***otx1b***	**30500**	**17**	**Exon 4**
***Gt(SA3-mCherry-T2A-CreERT2)tud38Gt***	***sox6***	**567154**	**4**	**Intron 4**

Full names of functional CreER^T2^-driver lines are designated as transgenic lines according to ZFIN nomenclature. Information of the gene trap integrations are shown with gene names, NCBI gene ID, chromosomal insertion (linkage group) and the insertion site relative to the gene architecture.

### Expression profile of tissue-specific CreER^T2^-driver lines

**Fig 5:**
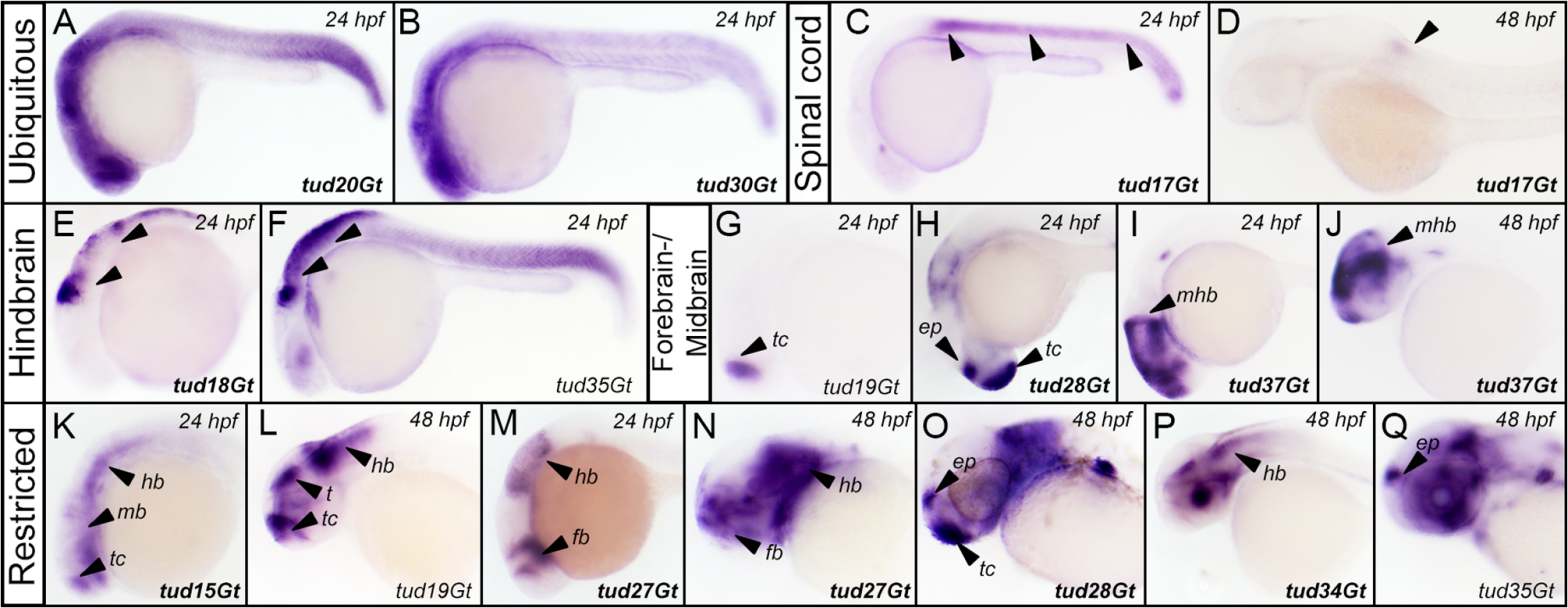
CreER^T2^-driver lines expressing in the embryonic neural tube. (A, B) Pan-neural expression of CreER^T2^ revealed by *in situ* hybridization in tud20Gt and tud30Gt at 24 hpf. (C,D) Expression of CreER^T2^ in the spinal cord in tud17Gt at 24 and 48 hpf. (E,F) Hindbrain expression of CreER^T2^ in tud18Gt and tud35Gt at 24 hpf. (G-J) Fore-/Midbrain expression and (K-Q) other restricted patterns of CreER^T2^ in tud15Gt, tud19Gt, tud27Gt, tud28Gt, tud34Gt, tud35Gt and tud37Gt at 24 and 48 hpf, respectively. Bold letters indicate CreER^T2^-driver lines with known gene trap integrations. (*See text for detailed description of expression patterns*.*);* ep: epiphysis; fb: forebrain; hb: hindbrain; mb: midbrain; mhb: mid-hindbrain-boundary; t: tectum; tc: telencephalon.

**Fig 6:**
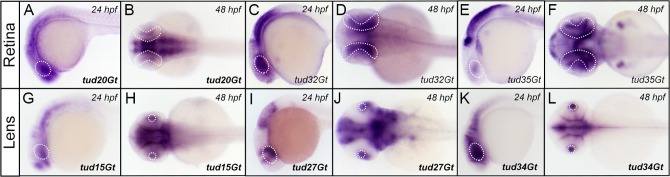
CreER^T2^-driver lines expressing in the embryonic eye. (A-F) Expression of CreER^T2^ in the developing retina in tud20Gt, tud32Gt and tud35Gt at 24 and 48 hpf, respectively. (G-L) CreER^T2^ is expressed in the developing lens in tud15Gt, tud27Gt and tud34Gt at 24 and 48 hpf, respectively. White dotted circles mark the retina (A-F) and lens (G-H). Bold letters indicate CreER^T2^-driver lines with known gene trap integrations. (*See text for detailed description of expression patterns*.*)*

**Fig 7:**
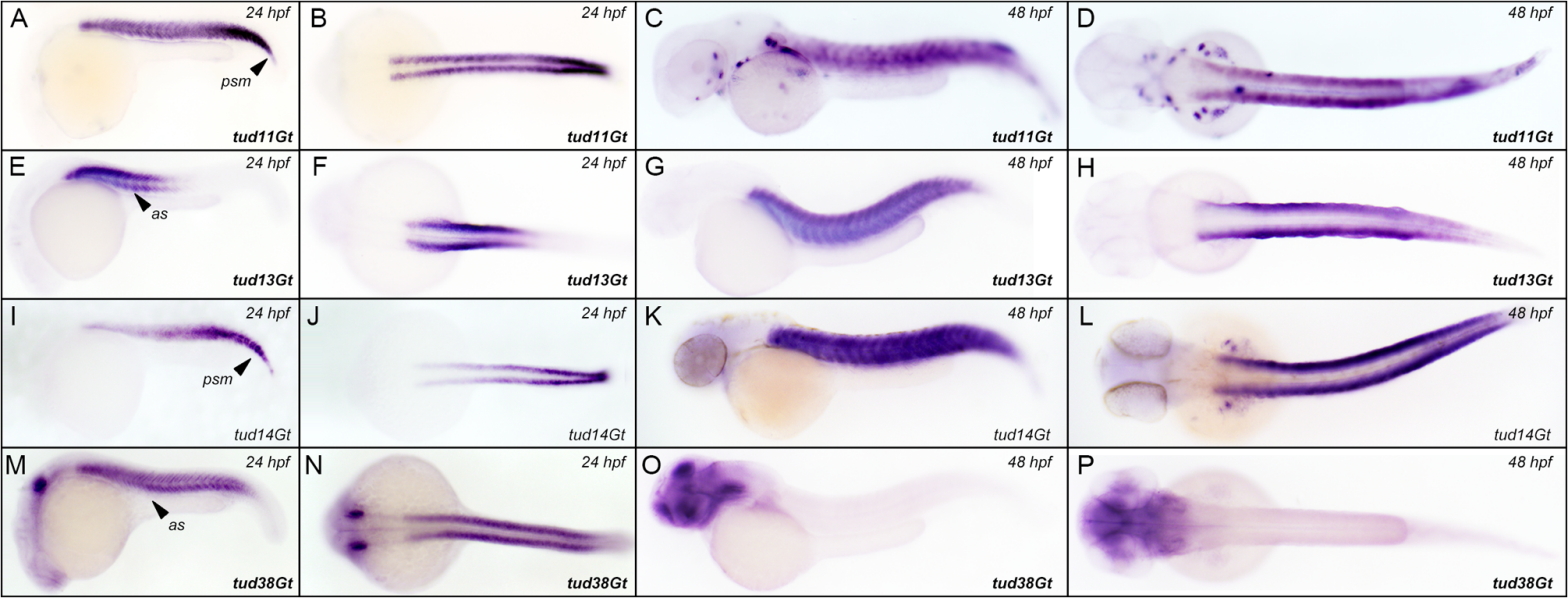
CreER^T2^-driver lines expressing in the somites. Expression of CreER^T2^ in (A-D) tud11Gt, (E-H) tud13Gt, (I-L) tud14Gt and (M-P) tud38Gt at 24 and 48 hpf.; Bold letters indicate CreER^T2^-driver lines with known gene trap integration. (*See text for detailed description of expression patterns*.*)* as: anterior somites; psm: presomitic mesoderm.

**Fig 8:**
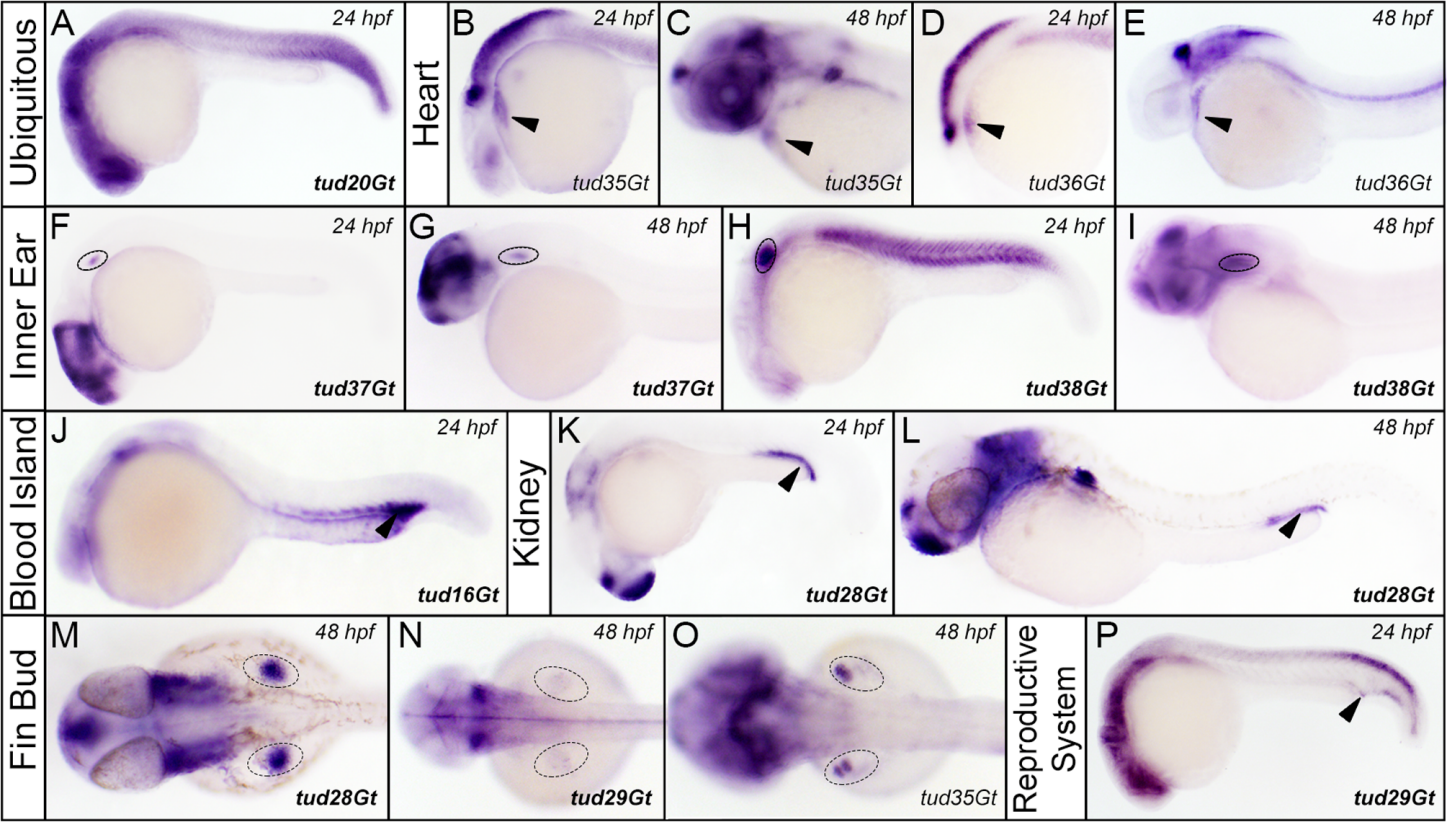
CreER^T2^-driver lines expressing in various embryonic tissues. (A) Ubiquitous expression of CreER^T2^ in tud20Gt at 24 hpf. (B-E) CreER^T2^ is expressed in the developing heart in tud35Gt and tud36Gt at 24 and 48 hpf. (F-P) Expression of CreER^T2^ can be detected in the anlagen of (F-I) the inner ear in tud37Gt and tud38Gt (J) blood island in tud16Gt, (K,L) kidney in tud28Gt (M-O) fin buds in tud28Gt, tud29Gt and tud35Gt and (P) the reproductive system in tud29Gt at 24 and 48 hpf. Bold letters indicate CreER^T2^-driver lines with known gene trap integrations. *(See text for description of expression patterns*.*)*

In order to determine the embryonic CreER^T2^ expression profile in more detail CreER^T2^ *in situ* hybridization (ISH) at 24 and 48 hpf was performed. This analysis corroborated that CreER^T2^ is expressed under the control of various endogenous promoters in various tissues, which were categorized using the “Phenotype Attribute and Trait Ontology” (PATO)-compliant terms to describe expression patterns with respect to anatomic regions [61], such as neural tube (Fig 5), eye (Fig 6), somites (Fig 7), fin bud, inner ear, ubiquitous, heart, blood island, reproductive system or kidney (Fig 8). To analyze whether gene trap integrations indeed resemble the endogenous gene transcripts, ISH was performed for both CreER^T2^ and respective endogenous genes. For example the insertion of the mCT2aC cassette into the *orthodenticle homolog 1b* (*otx1b)* locus in tud37Gt shows that CreER^T2^ expression faithfully recapitulates the endogenous *otx1b* expression. Expression of both CreER^T2^ and *otx1b* in tud37Gt transgenics or *otx1b* in wild-type siblings is restricted to the fore- and midbrain, as well as the developing inner ear at 24 and 48 hpf (S1 Fig). Another example is the insertion of the mCT2aC cassette into the *kelch-like 17 (klhl17)* locus in tud15Gt. Expression was observed in specific regions of the neural tube for CreER^T2^ transcripts of tud15Gt and *klhl17* transcripts in wild-type siblings (data not shown). Thus, we conclude that *pTol-SA*_*x*_ *-mCT2aC* gene trap integrations are able to report the endogenous expression pattern of trapped genes.

### CreER^T2^-driver lines expressing in the developing neural tube

24 functional CreER^T2^-driver lines show expression in the developing neural tube. Examples are depicted in Fig 5 with respect to different categories including ubiquitous expression in the neural tube (Fig 5A and 5B), spinal cord (Fig 5C and 5D), but also expression patterns restricted to the hindbrain (Fig 5E and 5F), fore- and midbrain (Fig 5G–5J), as well as other restricted neuronal patterns (Fig 5K–5Q). Broad expression in the neural tube is exemplarily shown in tud20Gt and tud30Gt at 24 hpf (Fig 5A and 5B) where integration of the mCherry-T2a-CreER^T2^ (mCT2aC) cassette occurred into the uncharacterized si:ch73-248e17.1 locus and the *serine/arginine-rich splicing factor 1b (srsf1b)* gene, respectively. Expression of CreER^T2^ in the entire spinal cord was detected in tud17Gt at 24 hpf (Fig 5C), which becomes restricted to the anterior spinal cord at 48 hpf (Fig 5D). Mapping of this insertion revealed integration of the mCT2aC cassette into the *homeobox B1b (hoxb1b)* locus, which was recently reported to control cell division, cell shape and microtubule dynamics during neural tube morphogenesis in zebrafish [62]. CreER^T2^-driver line tud18Gt shows expression in the hindbrain region at 24 hpf and was mapped to the *muscle segment homeobox C (msxc)* locus which has been previously described to be expressed in the hindbrain of early embryos [63]. Strong hindbrain expression was also detected in tud35Gt at 24 hpf (Fig 5F), which disperses into the fore- and midbrain at later stages (Fig 5Q). Unfortunately, integration mapping of this line was inconclusive. Restricted telencephalic expression was detected in tud19Gt in 24 hpf old embryos (Fig 5G), which expands into the mid- and hindbrain at 48 hpf (Fig 5L). Strong expression of CreER^T2^ was observed in the epiphyseal region as well as in the telencephalon in tud28Gt at 24 hpf with weaker expression in the hindbrain (Fig 5H). At 48 hpf, strong expression in the forebrain and epiphysis is maintained but hindbrain expression has significantly increased (Fig 5O). Insertion mapping revealed integration into the si:ch211-263k4.2 *(novel protein similar to H*.*sapiens PRDM16*, *PR domain containing 16)* locus. Another interesting CreER^T2^-driver line is represented by tud37Gt, where gene trapping has occurred into the *orthodenticle homolog 1b* (*otx1b)* locus. Expression is restricted to the fore- and midbrain abutting the mid-hindbrain-boundary (mhb) (Fig 5I and 5J). This pattern is consistent with previous reports detecting *otx1b* transcripts at high levels in a triangular patch already at mid-gastrula stage, which gives rise to fore- and midbrain structures [64]. Other restricted expression patterns include tud15Gt (Fig 5K), where gene trapping has occurred into the *kelch-like family member 17* (*klhl17)* gene, which is also known as *actinfilin*. Interestingly, expression analysis in rat brain indicated *actinfilin* to be expressed in neurons of most brain regions [65]. In addition, tud27Gt displays expression in the fore- and hindbrain, excluding the midbrain region at 24 and 48 hpf (Fig 5M and 5N). Molecular mapping revealed integration of the mCT2aC cassette into the *eph receptor A7 (epha7)* locus. In tud34Gt CreER^T2^ expression is present in the entire brain, excluding the telencephalon and tectum at 48 hpf (Fig 5P). Molecular mapping revealed integration into *tyrosine 3-monooxygenase/tryptophan 5-monooxygenase activation protein*, *beta polypeptide a (ywhaba)*.

### CreER^T2^-driver lines expressing in the developing eye

16 functional CreER^T2^-driver lines show expression in the embryonic eye, including the developing retina (Fig 6A–6F) and lens (Fig 6G–6L). Retinal expression is exemplarily shown in transgenic lines tud20Gt (Fig 6A and 6B), tud32Gt (Fig 6C and 6D) and tud35Gt (Fig 6E and 6F). CreER^T2^ is expressed broadly in the optic cup at 24 hpf in tud20Gt (Fig 6A) and tud32Gt (Fig 6C), whereas CreER^T2^ expression is restricted to a subdomain in tud35Gt (Fig 6E). At 48 hpf, CreER^T2^ transcripts are detected in a small region of the retina in tud20Gt (Fig 6B). Broader retinal expression is observed in tud32Gt (Fig 6D) and tud35Gt (Fig 6F). Mapping of these lines revealed either integration into an uncharacterized gene locus (tud20Gt) or remained inconclusive (tud32Gt, tud35Gt). In addition, we observed CreER^T2^-driver lines expressing in the embryonic lens, which develops at 30 to 36 hpf. Fairly broad CreER^T2^ expression in the eye field at 24 hpf was detected for all lines shown (Fig 6G, 6I and 6K). At 48 hpf expression was observed in both lens and retina for tud15Gt (Fig 6H). Interestingly, CreER^T2^ expression in tud27Gt at 48 hpf was restricted to the lens with adjacent expression in the temporal retina (Fig 6J). Restricted lens expression was also detected in tud34Gt at 48 hpf. Insertion mapping of these CreER^T2^-driver lines revealed integration into *kelch-like 17 (klhl17)* (tud15Gt), *eph receptor A7 (epha7)* (tud27Gt) and *tyrosine 3-monooxygenase/tryptophan 5-monooxygenase activation protein*, *beta polypeptide a (ywhaba)* (tud34Gt).

### CreER^T2^-driver lines expressing in the developing somites

We observed somitic CreER^T2^ expression in 9 functional CreER^T2^-driver lines (Fig 7). tud11Gt shows broad somitic CreER^T2^ expression at 24 and 48 hpf (Fig 7A–7D), including the presomitic mesoderm (PSM) at 24 hpf (Fig 7A). Gene trap integration occurred into *protein tyrosine kinase 2aa (ptk2aa)*, a human ortholog of *Focal adhesion kinase 1 (Fak1)*. Recent lineage tracing data using tud11Gt revealed that early *ptk2aa* expressing cells give rise to scale structures in zebrafish [50]. CreER^T2^ expression restricted to anterior somites at 24 hpf was observed in tud13Gt (Fig 7E and 7F), which expands into the posterior somites at 48 hpf (Fig 7G and 7H). Insertion mapping revealed integration into *parvalbumin 1 (pvalb1)*, which is known to be expressed in zebrafish skeletal muscle [66]. In tud14Gt, CreER^T2^ expression gradually increases from anterior to posterior somites and the PSM (Fig 7I and 7J) at 24 hpf. In contrast, strong and robust somatic CreER^T2^ expression can be found at 48 hpf (Fig 7K and 7L). Gene mapping of tud14Gt, however, remained inconclusive. Interestingly, in tud38Gt CreER^T2^ somitic expression is detected only at 24 hpf but is completely absent at 48 hpf (Fig 7M–7P). Molecular mapping revealed integration of the mCT2aC cassette into *SRY-box containing gene 6 (sox6)*, which plays, amongst others, important roles in zebrafish muscle fibre type specification and differentiation [67].

### CreER^T2^-driver lines expressing in other embryonic tissues

CreER^T2^ expression was also detected in various other embryonic tissues such as the anlagen of the heart (Fig 8B–8E), inner ear (Fig 8F–8I), blood island (Fig 8J), kidney (Fig 8K and 8L), fin bud (Fig 8M–8O) or reproductive system (Fig 8P). Ubiquitous CreER^T2^ expression was observed in tud20Gt at 24 hpf (Fig 8A). We detected expression of CreER^T2^ in the embryonic heart in tud35Gt and tud36Gt at 24 as well as 48 hpf (Fig 8B–8E). Unfortunately, gene mapping of these CreER^T2^-driver lines remained inconclusive. Transgenic lines tud37Gt (Fig 8F and 8G) and tud38Gt (Fig 8H and 8I) showed transgene expression in the anlagen of the inner ear at 24 and 48 hpf. Whereas CreER^T2^ expression is restricted to ventral cells of the otic vesicle in tud37Gt, the transgene is expressed throughout the otic vesicle in tud38Gt. Gene trap integration in tud37Gt occurred into the *orthodenticle homolog 1b* (*otx1b)* locus, which is involved in normal development of the zebrafish inner ear [68]. Also in mouse expression of *otx1* was detected in specific subdomains of the inner ear such as the lateral canal, the ampulla or the cochlea [69]. In tud38Gt the mCT2aC cassette was trapped into the *sox6* locus, which also has been described in the otic vesicle during mouse inner ear development [70]. Integration of the mCT2aC cassette occurred into the *cdc42 effector protein 4-like* gene locus in tud16Gt. Interestingly, CreER^T2^ expression is detected in the embryonic blood island, also known as the caudal hematopoietic tissue at 24 hpf (Fig 8J). Expression of the mCT2aC cassette in the embryonic kidney was detected in tud28Gt at 24 and 48 hpf (Fig 8K and 8L). Additionally, we found six gene trap lines expressing CreER^T2^ in the fin bud (Fig 8M–8O). In tud29Gt (Fig 8N) and tud35Gt (Fig 8O) CreER^T2^ expression is observed in restricted domains of the developing fin bud at 48 hpf. In tud28Gt CreER^T2^ is expressed in a fairly broad expression domain of the fin bud at 48 hpf (Fig 8M). Gene trap integration in tud11Gt occurred into *protein tyrosine kinase 2aa (ptk2aa)*. Molecular identification of tud29Gt revealed integration into *coiled-coil domain containing 102A (ccdc102a)*. Interestingly, tud29Gt shows CreER^T2^ expression, amongst others, in the reproductive system.

Overall, to date 59 Cre/CreER^T2^-driver lines have been described (Table 1). Addition of 27 novel functional CreER^T2^-driver lines described here, increases the number of available lines significantly (> 30%), and hence these lines represent important new tools to study zebrafish development, homeostasis and regeneration.

### Phenotype analysis

**Fig 9:**
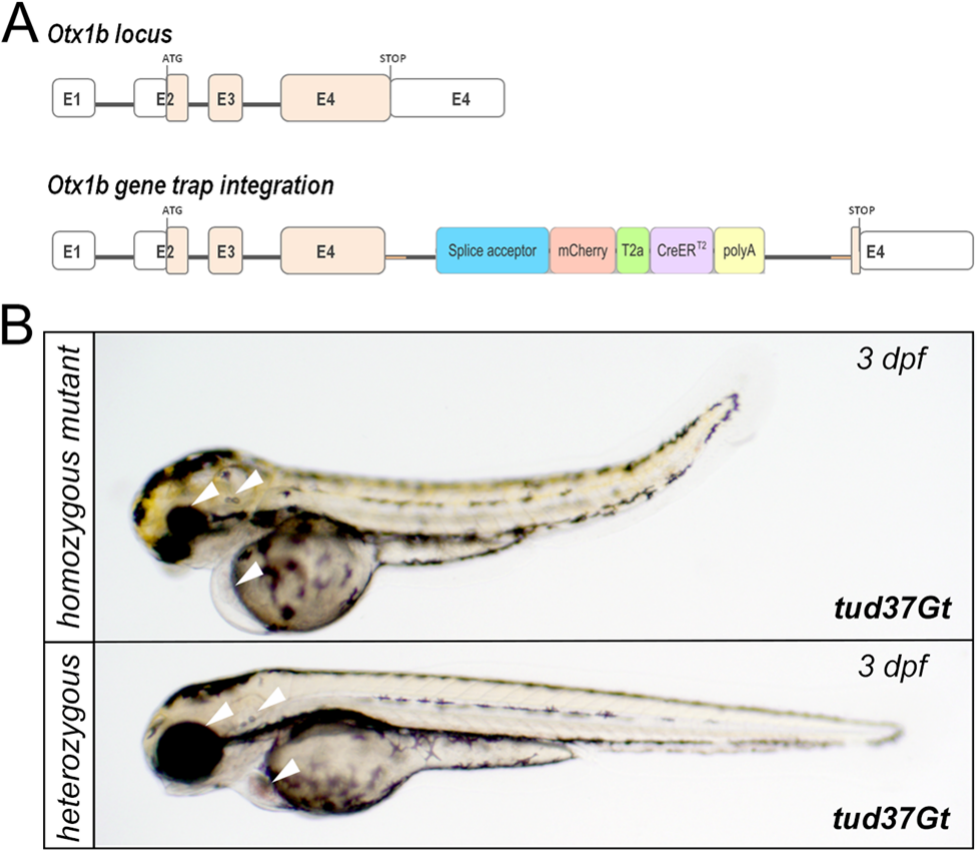
Gene trap insertion into the *otx1b* locus of tud37Gt. (A) Schematic drawing of the *otx1b* locus comprising of four exons (E1-E4) encoding a homeobox domain transcription factor. White boxes represent the 5’ and 3’ untranslated regions separated by the open reading frame in pink. The mCT2aC-cassette integrated into E4. (B) Bright field images of homozygous mutant tud37Gt embryos and heterozygous siblings. In comparison to heterozygous siblings, homozygous mutant tud37Gt embryos show defects in the developing eye, fore-/midbrain, ear and heart (white arrowheads) as well as a bend body shape.

Because of their ability to interfere with gene function, gene trap approaches have also been previously applied for insertional mutagenesis [44, 71] [72–74]. In most cases, vector insertion results in null or hypomorphic mutant phenotypes when inserted into the 5’ regions of a gene. To test if any insertion resulted in a developmental phenotype, all functional CreER^T2^-driver lines were inbred to obtain homozygous individuals which could be identified due to stronger mCherry fluorescence and were examined for morphological phenotypes in the first 5 days after fertilization. Of all 27 tested integrations, only homozygous tud37Gt animals showed an apparent phenotype. tud37Gt was mapped to the *otx1b* locus which is located on linkage group 17. The locus comprises four exons coding for a homeobox domain transcription factor (Fig 9A) [75]. Integration of the gene trap cassette occurred into exon four, resulting in a truncated Otx1b-mCherry fusion protein lacking parts of the transcription factor domain. Homozygous animals of tud37Gt display a variable phenotype affecting the development of eye, inner ear, forebrain, midbrain and heart (Fig 9B). Additionally, homozygous animals exhibit a bent body axis [76]. Analysis of more than 40 intercrosses revealed that the observed abnormalities are variable in strength, indicating additional genetic factors. Interestingly, knockdown of *otx1b* using antisense-morpholinos is less severe than the mutant phenotype observed in homozygous tud37Gt embryos/larvae [76, 77]. However, because the insertion occurred in the coding region of the transcription factor domain, the phenotype might also result from a dominant negative form of Otx1b protein.

Altogether, we conclude that integration of our *pTol-SA*_*x*_ *-mCT2aC* gene trap constructs rarely interferes with endogenous gene function and results only rarely in overtly apparent mutant phenotypes. In agreement with these results, previous reports in zebrafish have shown that integrations of other gene trap vectors such as the T2KSAG vector did not result in any mutant phenotypes [5].

## Discussion

Cre/loxP-technology has been successfully applied to dissect the zebrafish genome and genome-wide approaches have been conducted to create various Cre-effector lines [33–35]. However, currently the number of available cell- and tissue-specific Cre-driver lines to transactivate the before mentioned Cre-effector lines is limited [36]. By 2009, only 12 different Cre/CreER^T2^-driver lines had been published expressing Cre recombinase in a ubiquitous manner using the temperature inducible *hsp70l* promoter or tissue-specific promoter fragments driving expression in oocytes [78], pancreas [79], heart or neural tube [80]. In order to increase the existing pool of conditional Cre-driver lines we performed a genome-wide trapping screen using the mCherry-T2a-CreER^T2^ (mCT2aC) [21, 22] gene trap vector that yielded 27 new, fully functional CreER^T2^-driver lines expressing in various tissues in the developing zebrafish. The use of the mCT2aC cassette allows temporal control of Cre-mediated recombination using CreER^T2^. Initial conditional approaches in zebrafish have been carried out using Cre recombinase driven by the ubiquitous, temperature inducible *hsp70l* promoter [37, 81]. However, basal leakiness of the *hsp70l* promoter resulted in non-conditional Cre-mediated recombination even at permissive temperatures, limiting the usefulness of this approach [21, 22]. Non-conditional recombination has also been reported in cases when using CreER^T2^-constructs where high levels of CreER^T2^ might overwhelm the cellular machinery, preventing retention in the cytoplasm [24, 80]. To overcome this problem, Cre constructs fused with two LBD-domains have been generated [80, 82, 83]. However, although these constructs are more tightly regulated and display no non-conditional recombination, incomplete CreER^T2^-mediated recombination has been observed [80]. Therefore, Cre constructs with a single LBD-domain (CreER^T2^) currently provide the best option to achieve temporal control of Cre-mediated recombination. In addition to our gene trap lines, several promoter-fragment driven tissue-specific Cre/CreER^T2^-driver lines have been generated recently. To date, in total 63 Cre/CreER^T2^-driver lines have been described expressing either ubiquitously or in a tissue-specific manner [84] (data not shown). Thus, our new gene trap lines described here will increase this number by about 30% to 90 Cre/CreER^T2^-driver lines. The gene trapping approach using the *pTol-SA*_*x*_ *-mCT2aC* constructs provides an efficient method for generating numerous CreER^T2^-driver lines expressing in various tissues. Whereas promoter fragments often do not faithfully recapitulate the endogenous expression pattern [22, 85], gene trapping enables transgene expression driven by the endogenous promoter. Gene trapping is also faster and less expensive compared to the generation of Cre/CreER^T2^-driver lines using BAC (bacterial artificial chromosome) transgenesis [86]. Another promising tool to create Cre/CreER^T2^-driver lines will be the use of sequence specific transcription activator-like effector nucleases (TALENs) and the clustered regularly interspaced short palindromic repeats (CRISPR)/CRISPR-associated (Cas) 9 system (RNA-guided nucleases, RGNs). These endonuclease systems enable the targeted insertion of open reading frames [87] or whole plasmid vectors [88] [89], thus allowing specific knock-ins into any desired gene locus in zebrafish.

Although our gene trap approach offers several advantages for creating new CreER^T2^-driver lines, we also observed some difficulties. According to our functionality assay, only 64% of the selected CreER^T2^-driver lines showed successful recombination, whereas 36% remained non-functional. In general, gene trapping results in fusion transcripts of the N-terminal endogenous exons followed by the open reading frame of the gene trap vector. Consequently, signal sequences encoded by the N-terminus are included into the fusion protein and result in sorting of the fusion protein to various intracellular compartments. The viral T2a peptide of the mCT2aC gene trap cassette mediates cleavage only after translation [90]. Hence, cleavage of CreER^T2^ from the mCherry tagged truncated protein occurs only after a potential trapping event into an intracellular compartment and translocation of CreER^T2^ into the nucleus after TAM application is prevented, rendering it non-functional. Alternatively, internal ribosomal entry site (IRES) sequences could be used to achieve bicistronic expression of mCherry and CreER^T2^. IRES sequences have been successfully applied in mouse and recently also in zebrafish [91, 92]. In contrast to T2a peptides, IRES sequences allow independent protein translation of a bicistronic mRNA containing two open reading frames. As a result, subcellular localization would only affect the first, upstream cistron (mCherry), but not the second (CreER^T2^). However, non-zebrafish derived IRES-based gene expression is notoriously non-stoichiometric, creating disproportionate transgene translation levels [93], whereas the viral T2A peptide sequence allows the production of mCherry and CreER^T2^ proteins in equimolar ratios [90]. Recent identification of IRES sequences derived from zebrafish might overcome this problem by generating equal amounts of gene product from both cistrons [94]. Indeed, successful application of a zebrafish IRES for the generation of CreER^T2^-driver expressing in the heart has been reported recently by Jopling and colleagues [83]. The identification of the insertion site using 5’RACE was not always successful, which might be explained by low amounts of fusion transcript or by integration far away from the 5’ end. However, Trinh le and colleagues reported transgene mapping efficiencies up to 92% using 5’RACE [35]. Still, other methods might prove useful to detect gene trap integrations. For example splinkerette PCR has been successfully applied to assess the genomic integration of the FlipTrap vector [35, 95]. Furthermore linker-mediated PCR (LM-PCR) has been used to detect gene trap integrations on genomic level [34].

Previously, gene trap vectors have been reported to create mutant phenotypes upon insertion, [44, 71] [33, 72–74]. Due to an internal p(A) signal of the gene trap cassette, transcription of the endogenous gene is terminated, which leads to a truncated transcript. Generation of mutation-linked CreER^T2^-driver lines might be disadvantageous when studying biological processes, e.g. when studying lineage relationships. However, of all tested integrations only homozygous tud37Gt animals with an integration into the *otx1b* locus showed an overt phenotype, consistent with previous results showing that integrations of the gene trap vector T2KSAG vector did not result in mutant phenotypes [5]. This observation could be explained by weakness of the SA that might lead to alternative splicing of the gene trap cassette and hence, allow low level production of endogenous full-length transcript. For example, the application of another SA derived from the first intron of the carp *β-actin* gene shows high mutagenic potential [33, 74]. However, homozygous CreER^T2^ integrations are not required because single alleles are sufficient to elicit efficient CreER^T2^-mediated recombination. Thus, transactivation studies using CreER^T2^ gene trap lines can, in most cases, be expected not to interfere with insertion-related phenotypes.

The analysis of all selected CreER^T2^-driver lines produced during this gene trap screen, but also of other promoter-fragment based CreER^T2^-driver lines generated in our lab, have been summarized in an online database, the zebrafish CreZoo (http://crezoo.crt-dresden.de) [49]. CreZoo lines can be requested from the European Zebrafish Resource Center in Karlsruhe (EZRC) (http://www.ezrc.kit.edu/).

## Supporting Information

S1 Fig Insertion of the mCT2aC gene trap vectors recapitulates the endogenous gene expression pattern. Comparison of the endogenous *otx1b* expression in wild-type embryos with the CreER^T2^ expression pattern in tud37Gt embryos which has been mapped to the *otx1b* locus at 24 and 48 hpf. fb: forebrain; mb: midbrain; ov: otic vesicle.
(TIF)
