# Supplementary figures and images for: Isolation of Novel CreERT2-Driver Lines in Zebrafish Using an Unbiased Gene Trap Approach
Source: PLoS One. 2015 Jun 17;10(6):e0129072. doi: 10.1371/journal.pone.0129072 (PMC4471347; doi:10.1371/journal.pone.0129072)

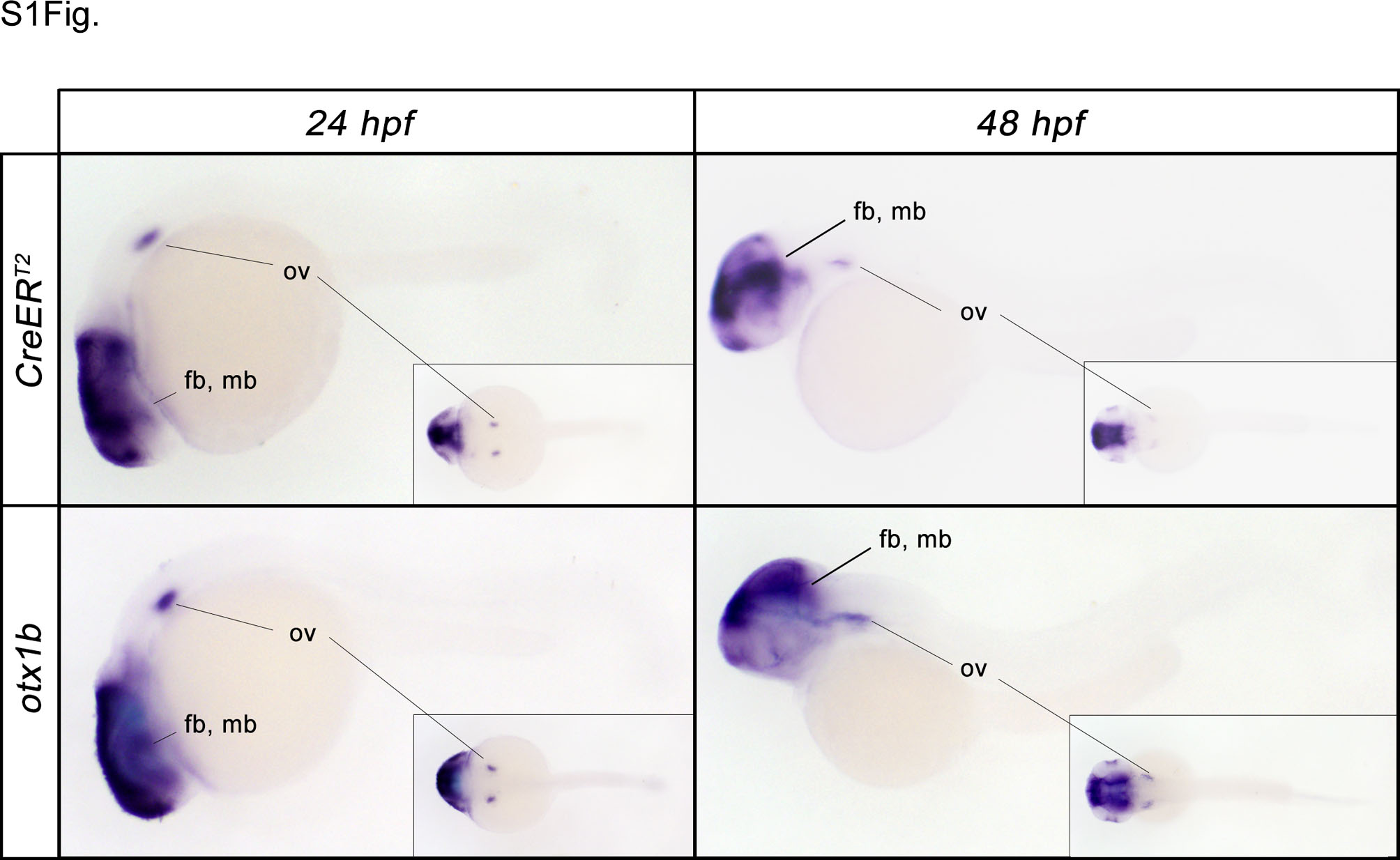

Supplement: S1 Fig — Comparison of the endogenous otx1b expression in wild-type embryos with the CreERT2 expression pattern in tud37Gt embryos which has been mapped to the otx1b locus at 24 and 48 hpf. fb: forebrain; mb: midbrain; ov: otic vesicle. (TIF) [file pone.0129072.s001.tif]
